# Meeting Cholera's Challenge to Haiti and the World: A Joint Statement on Cholera Prevention and Care

**DOI:** 10.1371/journal.pntd.0001145

**Published:** 2011-05-31

**Authors:** Paul Farmer, Charles Patrick Almazor, Emily T. Bahnsen, Donna Barry, Junior Bazile, Barry R. Bloom, Niranjan Bose, Thomas Brewer, Stephen B. Calderwood, John D. Clemens, Alejandro Cravioto, Eddy Eustache, Gregory Jérôme, Neha Gupta, Jason B. Harris, Howard H. Hiatt, Cassia Holstein, Peter J. Hotez, Louise C. Ivers, Vanessa B. Kerry, Serena P. Koenig, Regina C. LaRocque, Fernet Léandre, Wesler Lambert, Evan Lyon, John J. Mekalanos, Joia S. Mukherjee, Cate Oswald, Jean-William Pape, Anany Gretchko Prosper, Regina Rabinovich, Maxi Raymonville, Jean-Renold Réjouit, Laurence J. Ronan, Mark L. Rosenberg, Edward T. Ryan, Jeffrey D. Sachs, David A. Sack, Claude Surena, Arjun A. Suri, Ralph Ternier, Matthew K. Waldor, David Walton, Jonathan L. Weigel

**Affiliations:** 1 Department of Global Health and Social Medicine, Harvard Medical School, Boston, Massachusetts, United States of America; 2 Division of Global Health Equity, Brigham and Women's Hospital, Boston, Massachusetts, United States of America; 3 Partners In Health, Boston, Massachusetts, United States of America; 4 Zanmi Lasante (Partners In Health), Cange, Haiti; 5 Harvard School of Public Health, Boston, Massachusetts, United States of America; 6 Global Health Program, Bill & Melinda Gates Foundation, Seattle, Washington, United States of America; 7 Department of Medicine, Harvard Medical School, Boston, Massachusetts, United States of America; 8 Department of Microbiology and Molecular Genetics, Harvard Medical School, Boston, Massachusetts, United States of America; 9 Division of Infectious Diseases, Massachusetts General Hospital, Boston, Massachusetts, United States of America; 10 International Vaccine Institute, Seoul, Korea; 11 International Centre for Diarrhoeal Disease Research (ICDDR,B), Dhaka, Bangladesh; 12 Department of Pediatrics, Harvard Medical School, Boston, Massachusetts, United States of America; 13 American Society of Tropical Medicine & Hygiene, Deerfield, Illinois, United States of America; 14 Department of Microbiology, Immunology and Tropical Medicine, The George Washington University, Washington, D.C., United States of America; 15 PLoS Neglected Tropical Diseases, San Francisco, California, United States of America; 16 Sabin Vaccine Institute, Washington, D.C., United States of America; 17 Center for Global Health, Massachusetts General Hospital, Boston, Massachusetts, United States of America; 18 Center for Global Health, Weill Cornell Medical College, New York, New York, United States of America; 19 GHESKIO Centre, Port-au-Prince, Haiti; 20 The Task Force for Global Health, Decatur, Georgia, United States of America; 21 Tropical & Geographic Medicine Center, Massachusetts General Hospital, Boston, Massachusetts, United States of America; 22 The Earth Institute, Columbia University, New York, New York, United States of America; 23 Department of International Health, Johns Hopkins University Bloomberg School of Public Health, Baltimore, Maryland, United States of America; 24 Haitian Medical Association, Port-au-Prince, Haiti; 25 Channing Laboratory, Brigham and Women's Hospital/Harvard Medical School and HHMI, Boston, Massachusetts, United States of America; University of Tennessee, United States of America

**Executive Summary**

This joint statement argues for a comprehensive, integrated cholera response in Haiti. The cholera epidemic in Haiti is particularly devastating because of the vulnerability of Haiti's population after the January 12, 2010, earthquake, the long-standing weakness of its health, water, and sanitation systems, and the observed virulence of the El Tor hybrid strain. From October 19, 2010—when the first cases were confirmed in the National Public Health Laboratory—to April 4, 2011, 274,418 cases of cholera and 4,787 deaths related to cholera had been reported across all ten departments of Haiti [1].

The Haitian Ministère de la Santé Publique et de la Population (MSPP, the Ministry of Health) and the Direction Nationale de l'Eau Potable et de l'Assainissement (DINEPA, the government body charged with water and sanitation) have, with the support of many nongovernmental and international groups, made great strides against cholera. Case-fatality rates have dropped to 2.1% from 7% at the outset of the epidemic (and up to 10% in certain regions); incidence has also declined across Haiti, according to recent reports [1]. But fewer cases in the dry season (November–April) should not lead to complacency: seasonal variation is expected in epidemics of waterborne disease. Some have raised doubts about the sustainability of free water distribution within internally displaced persons (IDP) camps. But we believe that such efforts are an essential service that has contributed to the relatively few cases of cholera in the camps (as compared to other urban and rural areas).

Given the likelihood of case resurgence and endemicity of cholera in Haiti, this document argues for a comprehensive, integrated strategy for cholera prevention and care in Haiti. We must reduce suffering and preventable death in the short term, and we must build effective water, sanitation, and health delivery infrastructure to fortify Haiti against cholera and other diseases of poverty in the long term.

The document identifies three principal goals. First, we must continue aggressive case finding and scale up treatment efforts, including oral rehydration therapy, intravenous rehydration, antibiotic therapy (for moderate and severe cases), and complementary supplementation with zinc and vitamin A. Second, we must shore up Haiti's water infrastructure by building systems for consistent chlorination and filtration at public water sources and by distributing point-of-use water purification technologies. We must also strengthen sanitation infrastructure by improving and expanding waste management facilities (such as sewage systems and latrines) and waste monitoring. Third, we must link prevention to care by bolstering surveillance, education campaigns (about hand-washing, for example), and water, sanitation, and hygiene (WASH) efforts. Prevention must also include advocacy for scaled-up production of cholera vaccine and the development of a vaccine strategy for Haiti. A vaccination campaign should be implemented if adequate vaccine and resources can be mobilized without undermining efforts to treat acutely ill patients or strengthen water and sanitation infrastructure.

This document identifies key challenges and outlines the components of a comprehensive cholera response to aid medical and public health practitioners in Haiti and elsewhere. With leadership from the Haitian government, we must work together to bolster responses to the acute problem of cholera today and strengthen Haiti's health, water, and sanitation infrastructure to prevent similar outbreaks in the future.

## Introduction

### Cholera in Haiti: Acute-on-Chronic

Long before the devastating earthquake on January 12, 2010, Haiti struggled beneath the burdens of intractable poverty and ill health. The poorest country in the Western Hemisphere, Haiti also faces some of the highest rates of maternal and infant mortality—widely used indicators of the robustness of a health system—in the world ([S1] in Text S1; [2], [3]). The October 2010 cholera outbreak is the most recent of a long series of affronts to the health of Haiti's population; it is yet another acute symptom of the chronic weakness of Haiti's health, water, and sanitation systems.

Water and sanitation conditions highlight these systemic weaknesses. In 2002, Haiti ranked last out of 147 countries for water security [4], [5]. Before the earthquake struck, only half of the population in the capital, Port-au-Prince, had access to latrines or other forms of modern sanitation, and roughly one-third had no access to tap water [6]. Across the country, access to sanitation and clean water is even more limited: only 17% of Haitians had access to adequate sanitation in 2008, and 12% received treated water [7]. Not surprisingly, diarrheal diseases have long been a significant cause of death and disability, especially among children under 5 years of age [6].

The cholera outbreak began less than a year after a 7.0-magnitude earthquake took the lives of more than 300,000 people and left nearly 1.5 million homeless [6]. Almost 1 million Haitians still live in spontaneous settlements known as internally displaced persons (IDP) camps [8]. While post-earthquake conditions in Haiti were ripe for outbreaks of acute diarrheal illness, cholera was deemed “very unlikely to occur” by the United States Centers for Disease Control and Prevention (CDC) and other public health authorities [9]. Cholera had never before been reported in Haiti [S2] [10], [11]; health providers were unprepared for an influx of patients presenting with acute watery diarrhea.

The cholera epidemic has been most severe in rural areas and large urban slums. Rural communities were charged with hosting hundreds of thousands of displaced people after the earthquake, placing greater demands on their already-scarce resources, including water. Surface water drawn directly from the source or piped from rivers and streams constitutes the principal supply of drinking water in rural Haiti. The lack of adequate piping, filtration, and water treatment systems (including chlorination) made these rural regions vulnerable to the rapid spread of waterborne disease. While most IDP camps have been supplied with potable water, large urban slums have had to rely on existing water sources—some of them containing *Vibrio cholerae—*and have therefore been vulnerable to rapid disease spread. Most slums also have poor sanitation infrastructure. Since the first cases were reported in Saint-Marc and Mirebalais, cholera has spread to every department in Haiti, and to other countries, too [S3] [12]–[14].

Public suspicion (ultimately validated by genomic sequence analyses [15]) of the strain's link to South Asia, home to a group of United Nations peacekeepers stationed in central Haiti, triggered blame and violence that interfered with response efforts. As we have learned from the global AIDS pandemic and other infectious disease epidemics, cycles of accusation can continue for years, diverting attention and resources from the delivery of care and prevention services [16]. Systemic problems that brought cholera to epidemic levels in Haiti will (unless addressed) continue to facilitate its spread. As a disease of poverty, cholera preys upon the bottom of the social gradient; international trade, migration, and travel—from South Asia or elsewhere—open direct channels for pathogens that follow social fault lines.

### The Epidemiology of Cholera in Haiti

*V. cholerae* is a Gram-negative, rod-shaped, waterborne bacterium that causes acute watery diarrhea. A confirmed case requires laboratory analysis by culture of *V. cholerae.* Cholera causes rapid dehydration and electrolyte imbalances, and leads to death in up to 50% of untreated cases [17].

Cholera is endemic to Asia and Africa, with recent outbreaks in Angola, Ethiopia, Zimbabwe, Pakistan, Somalia, Sudan, and northern Vietnam. From 1991 to 1994, Latin America experienced a multi-country epidemic of more than 1,000,000 cases and 10,000 deaths. The disease was not reported in the Caribbean, however, until the current epidemic in Haiti. While patients began presenting with severe watery diarrhea as early as October 13, 2010, the first laboratory-confirmed cholera cases (from the Artibonite Department) were documented on October 19, 2010 [S4]. Within days, the National Public Health Laboratory (LNSP) in Haiti had isolated *V. cholerae* serogroup O1 of the El Tor biotype as the cause of these cases of diarrhea, dehydration, and death [12].

Cholera is expected to be most severe among immunologically naïve populations [18], and the Haitian outbreak exhibited an initial 7% case-fatality rate—among the highest recorded rates in recent history [S5] [18], [19]. There were more than 2,000 reported cholera-related deaths in 40 days [S6] [20]—nearly half the number of total deaths registered in Zimbabwe's year-long epidemic [S7] [21]—and these figures are likely underreported in many rural areas [S8] [22].

As of April 4, 2011, the MSPP reported 274,418 cases of cholera and 4,787 deaths attributed to cholera across all ten of Haiti's departments. The overall observed case-fatality rate was 1.7% [1]. However, cumulative case-fatality rates range from 0.8% to 7.7% across regions (see Table 1), revealing both the geographic disparities that have patterned the epidemic and the great strides in cholera care in certain regions [23].

**Table 1:** Geographic variations in cumulative cases and fatalities (October 20, 2010–April 8, 2011) [24].

Department	Total Cases	Hospitalized Case Fatalities	Non-Hospitalized Case Fatalities	Case-Fatality Rate
Artibonite	66,285	568	322	1.3%
Centre	24,782	201	182	1.5%
Grande Anse‡	15,351	343	497	5.3%
Nippes	3,618	62	93	4.2%
Nord‡	27,930	590	45	2.3%
Nord Ouest	16,410	176	72	1.5%
Nord Est	11,833	117	150	2.2%
Ouest*	26,404	255	121	1.4%
Port-au-Prince**	67,579	416	141	0.8%
Sud‡	13,791	181	55	1.7%
Sud Est	3,033	76	172	7.7%

**‡:** Excluding Port-au-Prince.

*Incomplete data available.

**Port-au-Prince: Carrefour, Cité Soleil, Delmas, Kenscoff, Petion Ville, Port-au-Prince, et Tabarre.

**Figure 1:**
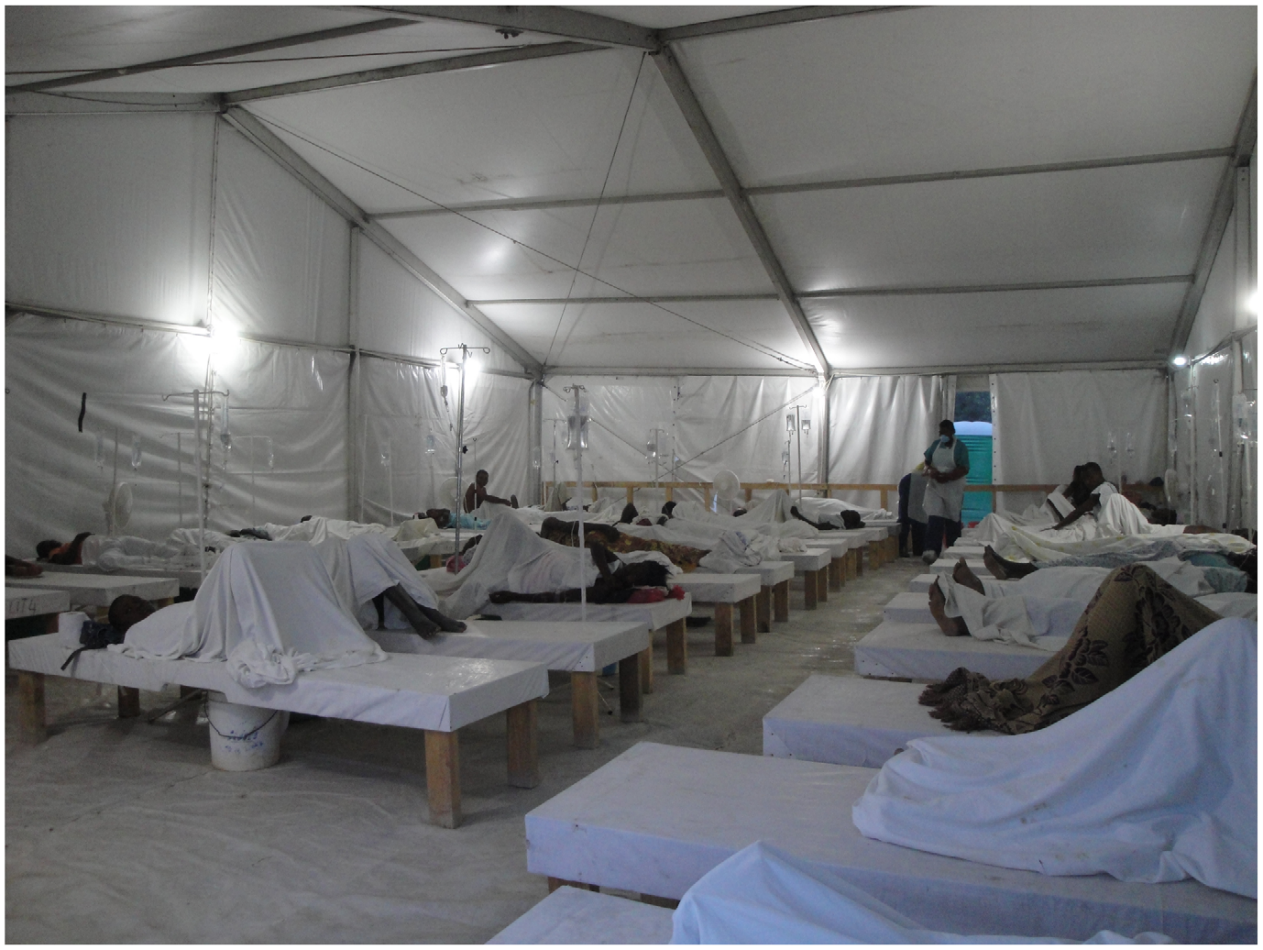
Cholera treatment center, Mirebalais, Haiti, December, 2010. Photo credit: Jonathan L. Weigel, Harvard Medical School and Partners In Health.

The reduction of case-fatality rates to under 2% reflects the implementation of rapid treatment and case management efforts (Figure 1). Yet as cholera continues to strain Haiti's health infrastructure, some predict an increase in overall mortality [20]. The majority of cholera-related deaths occur among patients who do not reach a hospital in time. Such deaths are likely underreported in official statistics. Sustaining the gains made in recent months will require close surveillance and rapid treatment if there are case resurgences.

During the first 2 months of the epidemic, cholera spread most rapidly in the central and northern parts of the country [S9] [24]. National elections and ensuing riots occurred in late November 2010, preventing many patients in Port-au-Prince from reaching health centers in time, if at all. The Artibonite Department, where the first cases were officially documented, reported the most cases: over 66,000 by April 8, 2011 [23].

Overall incidence in IDP camps has remained low. (Some camps have reported mere dozens of cases.) This achievement is chiefly thanks to the work of the Ministère de la Santé Publique et de la Population (MSPP), Direction Nationale de l'Eau Potable et de l'Assainissement (DINEPA), and many partnering institutions participating in water, sanitation, and hygiene (WASH) programs. Such efforts have, among other things, made safe drinking water available—mostly for free—to camp-dwellers. Nonetheless, poor sanitation conditions among the camps could lead to rapid disease transmission if access to clean water declines, and recent doubts about the “sustainability” of free water distribution threaten to undermine such access.

Compared with previous strains of *V. cholerae*, some evidence suggests that the hybrid El Tor strains in Haiti cause more asymptomatic cases, persist longer in the environment, and exist in higher concentrations of bacteria in feces (including in asymptomatic cases) [S10] [25], [26]. Even though some data suggest a lower ratio of symptomatic to asymptomatic cases, a resurgence of cases is likely if surveillance, prevention, and treatment efforts are not scaled up.

If past is prologue, there is little reason to believe that the “Haitian” cholera epidemic will remain strictly Haitian for long. Haiti is part of a web of global connections, and pathogens like *V. cholerae*, HIV, or *Mycobacterium tuberculosis* travel freely throughout this web. Cases traceable to the Haitian outbreak have already been reported in the Dominican Republic, Venezuela, Florida, and Massachusetts [S11] [12]–[14]. These introductions have not led to widespread disease and are not expected to do so in areas with adequate water and sanitation systems. But in other neighboring countries in the Caribbean and Latin America that lack these systems, the threat of a multi-country epidemic—like the Latin American epidemic in the early 1990s—is real.

### A Comprehensive, Integrated Strategy for Cholera Prevention and Care

A history of poverty, natural disaster, neglected public water and sanitation systems, and under-resourced health infrastructure has magnified the impact of cholera in Haiti. Some have called these conditions a “perfect storm for a massive epidemic of cholera” [S12] [6]. Responding to cholera today must address the same long-standing challenges that have prevented Haiti's poor from accessing clean water, adequate food, and decent health services for decades. An effective strategy for cholera prevention and care must continue aggressive case-finding and treatment efforts, shore up Haiti's water and sanitation systems, expand the availability of prevention services, and coordinate these cholera-specific interventions to strengthen Haiti's health system.

Ongoing efforts led by the Haitian government and local and international relief teams have already reduced incidence and case-fatality rates across the country. But many medical and public health teams lack the tools of their trade—including oral rehydration salts (ORS), intravenous solution, antibiotics, vaccines, soap, cholera cots, ambulances—and the rainy season (May–October) is approaching. We must move together to marshal the tools needed to control the epidemic and to fortify Haiti against cholera for the long term. Divisions over the respective roles of prevention and care are as senseless as those over the use (or lack thereof) of vaccines and antibiotics; in the face of cholera's challenge to Haiti and the world, we can accept nothing less than complementary and comprehensive prevention and care.

## GOAL 1. Bolster Case Finding and Treatment

Considering the observed virulence of this El Tor strain and the fragility of the Haitian health system, aggressive case finding and treatment must be scaled up. Treatments are inexpensive, effective, and can be coordinated through existing referral and communication systems. Although a network of treatment sites is in place, too many patients are unable to get the medical care they need or arrive at cholera treatment centers in time. Not only do these individuals face the debilitating symptoms of cholera (sometimes ending in death), they continue to spread infectious organisms. We must expand case finding, patient transport, and treatment and integrate such efforts into the public health system to facilitate responses to future cholera epidemics.

### Aggressive Case Finding and Efficient Transport

Most of the hundreds of thousands of patients who have received care to date have not reached cholera treatment sites as a result of case-finding efforts; they have fallen ill and been brought in by their families, sometimes too late [27]. A mortality assessment by the CDC found that some patients in the Artibonite Department died as early as 2 hours after first exhibiting symptoms [12]. Early in the epidemic, it was estimated that 40% of cholera-related deaths occurred because patients did not arrive at treatment sites in time to receive sufficient care (Dr. Jean-William Pape, personal communication). Even when patients arrive for care early in the course of their sickness, many do not receive life-saving therapeutics like antibiotics due to limited supplies at treatment sites. Case-fatality rates of untreated cholera have been reported as high as 50%, but can be reduced to 1% with rapid and comprehensive treatment, as has been shown in some parts of Haiti after scaled-up treatment efforts [25].

However, patient transport and *in situ* care remains a bottleneck to scaling up cholera treatment, especially in rural areas. Ambulances are in short supply [27]. With more resources and training, community health workers could improve case finding and help overcome delivery challenges like patient transport.

### Making Treatment Substantial: From ORS to Antibiotics

Treating dehydration—with ORS and intravenous fluids, both inexpensive and deliverable *in situ*—is the key to saving lives. World Health Organization (WHO) guidelines provide a framework for managing cholera treatment based on case severity [28]. Over 80% of cholera patients can be treated with ORS alone [28], and yet almost 2 months into the epidemic, the CDC identified a sizeable gap in access to ORS [12]. This gap arose in spite of great strides against childhood diarrhea in Haiti (using ORS and other interventions effective in treating cholera) made by the MSPP, Cornell University, and others. These efforts have helped to bring down infant mortality rates by about two-thirds since 1980 [S13] [29]–[31]. In recent months, access to ORS has improved significantly, contributing to the observed decreases in case-fatality rates countrywide. Nonetheless, ensuring that ORS is readily available to all populations at risk of infection, along with developing ORS stockpiles for future outbreaks, remains a high priority.

But treatment cannot stop with rehydration: patients with moderate and severe disease can still die from persistent fluid loss while receiving ORS. These patients need intravenous rehydration and antibiotic therapy. In addition, innovative therapeutics like rice-based ORS, zinc supplements, vitamin A, and deworming agents should be integrated with existing treatment efforts. Most ORS is glucose-based, but some studies have found rice-based varieties to be more effective, particularly in reducing mean stool output [32], [33]. This formulation may also improve nutrition. Zinc supplements have been shown to reduce the duration of diarrhea, particularly in children, and are therefore recommended by the WHO for patients with most diarrheal diseases, including cholera [S14] [34]–[36]. A review of efficacy studies noted a 23% decrease in diarrhea-related mortality due to zinc treatment [37]. One study found that zinc treatment for children with cholera reduced vomiting, diarrhea output and duration, and hospital stay by 8 hours [36]. Vitamin A deficiency, sometimes caused or worsened by diarrheal diseases, can lead to eye lesions in young children. The WHO therefore recommends vitamin A supplements for all individuals with cholera, especially children [S15] [34]. Deworming agents and childhood vaccinations—readily deliverable in resource-poor settings and yet rarely available in Haiti—should also be integrated with cholera prevention and care. Such piggybacking interventions could decrease coinfection with other diseases of poverty that predispose children with cholera to poor treatment outcomes and reduce morbidity, mortality, and malnutrition among Haiti's population [34]. Each of these interventions is part of a comprehensive cholera care package.

### The Case for Antibiotics

Given Haiti's fragile health system and the observed virulence of the hybrid El Tor strain, there is growing consensus about the urgency of making antibiotic therapy available for all moderate and severe cases of cholera (all hospitalized patients). Policy has shifted in this direction: most global public health policymakers—including the WHO and the Pan American Health Organization (PAHO), CDC, MSPP, and the International Centre for Diarrhoeal Disease Research, Bangladesh (ICDDR,B)—now recommend antibiotics for all moderate and severe disease [38], [39]. But providers must be furnished with adequate supplies to meet these guidelines. To date, too few patients with moderate disease receive antibiotic treatment. We must move rapidly to expand the use of antibiotics for cholera treatment, which can shorten the severity and course of illness, reduce transmission, and lessen the burden on Haiti's health system.

First, antibiotics have been shown to shorten the duration of symptomatic cholera and therefore limit life-threatening dehydration. The current strain in circulation in Haiti is susceptible to doxycycline and azithromycin, resistant to nalidixic acid and sulfisoxazole (a marker for co-trimoxazole resistance), and has reduced susceptibility to ciprofloxacin [12]. Azithromycin and doxycycline are effective in treating severe cholera cases. Azithromycin reduces the duration and volume of diarrhea and vomiting in strains with reduced susceptibility to ciprofloxacin [40]. Broader use of single-dose azithromycin or doxycycline in all hospitalized patients should be implemented immediately. Monitoring for antibiotic resistance on a monthly basis would help ensure that antibiotic use remains consistent with the antibiotic sensitivity of the current strain.

Second, antibiotic use brings public health benefits by reducing transmission. Antibiotic treatment can cut stool volume in half [40] and shorten the amount of time patients shed infectious organisms from several days to 1 day [40]. Such organisms exhibit a hyperinfectious quality 5–24 hours after output, elevating the risk of transmission to those living in city slums, IDP camps, and households, as well as others living in close proximity with cholera patients [41] [18]. In Bangladesh, it has been shown that 20%–30% of patients' household contacts develop symptoms of cholera within 10–21 days [42]. Therefore, antibiotics are a pillar of both prevention and treatment.

Third, expanding access to antibiotic treatment for all moderate and severe cases would mitigate the strain on the health system by decreasing the severity of illness and duration of inpatient care (therefore freeing hospital beds for other patients) [40]. Many treatment sites already have more patients than beds, intravenous hydration materials, and other critical supplies. Reduced demand for inpatient services would also make resources and staff available for other components of cholera prevention and care [S16].

In addition to providing antibiotic treatment for all moderate and severe cases, we suggest launching a closely monitored study about the efficacy of these drugs for mild cases and for prophylactic use among certain vulnerable populations, such as family members, health care workers, and cellmates of infected prisoners. Some evidence from past epidemics in Tanzania and Ecuador suggests that chemoprophylaxis may lead to bacterial resistance without compensatory gains in survival [43]. Other experiences indicate that closely monitored chemoprophylaxis can bring health benefits. For example, one project at the National Penitentiary of Port-au-Prince found that providing antibiotics (along with potable water, soap, and sanitation services) to prisoners sharing living quarters with cholera patients helped reduce incidence within the institution [44]. The risk of resistance means that we should avoid large-scale chemoprophylaxis campaigns until more research is conducted.

### Delivery Challenges to Cholera Care in Haiti

A triage system of independent treatment sites—cholera treatment centers (CTCs) and cholera treatment units (CTUs)—has been deployed in Haiti [28]. CTCs have an average capacity of 100–400 beds, whereas CTUs tend to have 15–20 beds and are often attached to existing health facilities [27]. By providing emergency care for cholera patients, CTUs allow hospitals and health centers to continue delivering normal health services. In addition, more than 900 oral rehydration points (ORPs) across the country help treat patients with mild disease [S17] [45]. The CTUs and ORPs serve as a first point of entry into the health system for individuals with severe watery diarrhea. Patients are then either stabilized and sent home or referred to CTCs [46]. A PAHO bulletin published on January 23, 2011, reported 85 CTCs and 129 CTUs throughout the country [47]. Although some facilities have recently been closed because of decreasing disease incidence, they may again be needed if there is a resurgence of cases in the coming months and years.

There are substantial delivery challenges in both urban and rural Haiti. On the one hand, densely populated urban areas, including slums and IDP camps, are a volatile setting for fecal–oral bacterial transmission. On the other hand, many populations in well-managed camps have better access to treated drinking water (often from a centralized, chlorinated source) [48], adequate sanitation, and medical care provided by humanitarian groups. Furthermore, the density of vulnerable populations facilitates case detection and rapid distribution of interventions, including education, oral rehydration, and vaccination. But not all camps are managed, and even those with decent sanitation and clean water are still temporary settlements; we cannot count on humanitarian groups to help provide these services indefinitely.

Rural regions, where the majority of cholera cases have been recorded, face many delivery challenges, including large distances between patients and treatment centers, poor infrastructure, inadequate transport services, and insufficient health personnel [S18]. Médecins Sans Frontières, an international nongovernmental organization (NGO) that has provided health care in rural Haiti for decades, has stressed that rural communities are at high risk of recurrent cholera outbreaks [49]. Scaled-up case finding and treatment are needed to ensure rapid access to care in rural areas [47], [50].

### Human Resources for Health: Community Health Workers as a Cornerstone of Care

One of the greatest obstacles to the effective delivery of health services in Haiti is the lack of health personnel. Haiti faced a shortage of health care workers prior to the cholera epidemic, and in early December 2010, PAHO called for an additional 350 doctors, 2,000 nurses, 2,200 support staff, and 30,000 community health workers (CHWs) to respond to the outbreak [51]. Encouraging gains have been made: the CDC has worked closely with the MSPP, ICDDR,B, and PAHO, for example, on a train-the-trainers program to teach health workers to prevent, identify, and treat cholera [52], [53]. “Master trainers” who have graduated from this program are assigned to departments to train other health staff at cholera treatment sites [S19] [12]. By the end of 2010, programs had been completed in nine of Haiti's ten departments, producing more than 500 graduates [54].

As the epidemic lingers in Haiti, CHWs should be the main line of defense against case resurgence. CHWs have been an integral part of effective community-based tuberculosis and HIV/AIDS prevention and care in Haiti [55]. They can be trained to diagnose cholera and initiate treatment (especially ORS) *in situ*, including in rural areas [20]. For example, the ICDDR,B's Cholera Outbreak Training and Shigellosis Program in Bangladesh demonstrated that CHWs can effectively administer ORS [56]. ICDDR,B materials are now available in Haitian Creole.

Training initiatives have contributed to declining incidence and case-fatality rates. Cholera management skills must now be integrated into the regular training package for all health workers.

## GOAL 2. Strengthen Water and Sanitation Systems

### A History of Water Insecurity

The cholera epidemic is a symptom of Haiti's long history of water insecurity.

Although some NGOs have put resources into local water projects over the years, private projects cannot replace a robust public sector water system [S20]. But political instability and crippling debt, among other factors, have kept the Haitian government from providing a safe supply of drinking water for its citizens. Almost a decade before the 2010 earthquake, Haiti was ranked last of 147 countries on the Water Poverty Index (a measure of water security) [4], [5], [57] and 101st out of 122 countries for water quality [58]. The percentage of Haitians with access to safe drinking water actually decreased 7% between 1990 and 2005 [59]; only 30% had access in 2004 [S21] [60]. The lack of modern sanitation further aggravates the situation: only 27% of the country had basic sewage in 2004 [61], [62]. Such underdevelopment fuels a vicious cycle of poverty, poor sanitation, water contamination, and ill health [S22] [59], [63], [64].

Haiti's fragile water infrastructure received another blow in the January 2010 earthquake. One month after the outbreak, 521 of 1,356 IDP camps listed by the United Nations (UN) shelter cluster had no water or sanitation services [20]. However, progress has been made: by the end of February 2011, IDP residents had an average of 17 liters of potable water per person per day, exceeding the Sphere Project 15-liter standard (unpublished 2011 data from the CDC) [65], [66]. This promising trend must be continued, and we must remain alert to disparities of access among and within camps. For example, some have suggested charging for drinking water within informal settlements and IDP camps on the grounds that free water distribution—a service that has been available in most camps, and one of the principal reasons why they have had low incidence of cholera—is not sustainable. A cost-recovery mechanism requiring payment for access was instead recommended [27]. But camp-dwellers have little (if any) income, most of which goes toward food and other basic needs. Anyone who has worked with Haiti's urban or rural poor would predict that this brand of “cost-recovery”—shifting the burden of payment onto the poorest people—will lead camp-dwellers to look elsewhere for water; but in post-earthquake Haiti, most other sources are not clean or cholera-free [59], [67].

### Water Treatment Systems

Current efforts to prevent transmission of cholera in Haiti must be continued at the same time that treatment and filtration systems at public water sources are strengthened in the long term. In the short term, point-of-use treatment and filtration technologies should also be rolled out to improve immediate access to clean water on a household level. Furthermore, working in conjunction with DINEPA and the Ministère des Travaux Publics Transports et Communications (MTPTC, Ministry of Public Works and Communication) would allow NGO projects to build the capacity of municipal water systems and therefore improve Haiti's long-term water security.

On the health provider end, ORS must be prepared and stored in sanitary conditions or it can do more harm than good. One study in Guinea-Bissau found that ORS prepared in an open tub was highly susceptible to bacterial contamination. Storing ORS in a narrow-mouthed spigot container and rinsing vessels with chlorinated water reduced such contamination [S23] [68]. Health workers at treatment sites across the country must have access to guidelines based on these findings.

On the household end, point-of-use water purification tools can help prevent diarrheal illness, even in settings of poverty and water insecurity. For example, household water treatment and safe storage (HWTS) technologies offer families an independent means of water treatment and storage, and they have been shown to reduce waterborne disease transmission—even during epidemics. Studies estimate 30%–40% reductions in diarrheal disease due to improved household drinking water quality at the point of use [S24] [69], [70]. The 2002 World Health Report highlights HWTS as the most cost-effective intervention for preventing diarrheal disease in a range of contexts [71]. Although sometimes construed as emergency interventions, point-of-use water treatment systems have also been found to contribute to long-term reductions in waterborne disease incidence [72].

Boiling and solar disinfection can be difficult to deliver in Haiti due to high costs and infrastructural constraints. Point-of-use filtration/disinfection units and sachets combining flocculation (physical filtration), chlorination, and safe storage are affordable, deliverable in resource-poor areas, and produce enough safe water for multiple households [S25] [73]–[76]. Flocculation-disinfectant powder sachets, which cost less than US$10 per year in most cases [S26] [77], have also proved useful in resource-poor settings [S27] [74], [78], though turbid water reduces their efficacy [S28]. Simple cloth filtration, shown to reduce waterborne disease transmission by up to 48% [S29] [79], can supplement sachet-based treatment to further decrease the likelihood of *V. cholerae* contamination [73].

Although household-based purification systems should be deployed widely, strengthening Haiti's public water system remains the best way to improve water security in the long term. As noted, 70% of Haiti's population lacked access to potable water before the earthquake [60]. But even this statistic may overstate access to improved water sources in Haiti because public systems are rarely available year round. The World Bank concluded that “in almost all urban areas water supply is intermittent”; in rural areas, supply is equally unpredictable, particularly during the dry season [80]. The lower Artibonite city of Saint-Marc offers one example of a public water system that, although long weakened, has been improved in recent years thanks to international and private sector collaboration (Dr. Charles Patrick Almazor and Inter-American Development Bank staff, personal communication). Only robust municipal water systems that are maintained and monitored by the MTPTC would safeguard access to clean water across Haiti.

Some have suggested that household-based interventions are more effective at preventing diarrheal disease than interventions at the source [S30] [70], [74], [81]. However, this is not an either/or scenario: both improved purification technologies within households and strengthening municipal water systems are essential components of water treatment. Support from NGOs and other international groups is needed in both the short- and long-term, but all such efforts must be coordinated by the MTPTC to develop the infrastructure and capacity of the public water system.

### Sanitation Tools

As noted, close patient contacts (in slums, IDP camps, prisons, households, and treatment sites, for example) are at particularly high risk of infection because of the hyperinfectious state exhibited by *V. cholerae* in excrement [42], [82], [83]. Though the lack of modern sanitation is a principal cause of fecal–oral bacterial transmission, few substantial sanitation projects have been launched in Haiti. At least four steps are needed to strengthen Haiti's sanitation system in the long term: systematic hand-washing with soap, improvement and installation of modern sewage systems and latrines, integration of waste monitoring with water surveillance, and improvement of cadaver management.

First, hand-washing with soap can decrease the risk of contracting most diarrheal diseases [S31] [84]. The WHO emphasizes that systematic hand-washing with soap before eating and handling food and after defecation remains one of the surest ways to prevent cholera transmission [85]. Studies estimate that proper hand-washing can reduce the risk of diarrheal illness by up to 47% [86]. Yet while soap costs about US$0.50 in Haiti, most Haitians live on less than US$1.25 per day, most of which goes towards basic needs such as food [87]. In the first months of the epidemic, the UN Office for the Coordination of Humanitarian Affairs (OCHA) estimated that 11,000 bars of soap would be necessary to cover certain priority IDP camps for 2 weeks [88]. However, 2 months after the onset of the epidemic, the UN shelter cluster response teams reported significant shortages of needed materials, including soap, water, purification tablets, and latrines [89]. More recently, OCHA projected that 4.5 million Haitians (3 million in the 30 main cities and towns and 1.5 million in rural areas) would benefit from WASH programs, and the low incidence of disease in the IDP camps is, in part, a testament to these efforts [89]. Nonetheless, soap distribution and hand-washing education efforts should be scaled up in vulnerable rural and urban areas.

Second, better sewage systems and latrines would help curb cholera transmission. Pit latrines are a good option for rural areas, as they would strengthen sanitation infrastructure. In the crowded and concrete streets of Port-au-Prince, above-ground sewage tanks are the best available short-term solution. As part of the city's reconstruction, however, permanent underground sewage systems must be put in place to decrease the likelihood of fecal–water contamination and transmission of waterborne disease [S32] [90].

Third, waste monitoring can decrease the spread of waterborne disease and also improve outbreak prediction. A study in Peru found that waste and water surveillance—including weekly sewage analysis for *V. cholerae* O1 and vibriophages—signaled a cholera outbreak 1 month in advance, facilitating the rapid implementation of prevention measures [S33] [91]. A similar approach should be explored in Haiti in conjunction with the MTPTC.

Fourth, safe disposal of cadavers must also be integrated into the broader waste management system. Cadaver management poses many challenges, including honoring cultural practices related to dying and death, disinfecting cadavers, and identifying burial sites that are acceptable to the community and will not contaminate the waterbed [S34] [92]. Stigma also prevents effective cadaver management: many funeral parlors refuse cadavers from patients who died of cholera, for example. Some efforts are underway to develop best practices for the safe disposal of cadavers. Organizations like the Haitian Group for the Study of Kaposi's Sarcoma and Opportunistic Infections (GHESKIO) are training funeral parlor staff to decontaminate and handle cadavers. Similar initiatives addressing the safe disposal of cadavers would help curb the spread of cholera in Haiti.

## GOAL 3. Link Prevention to Care

### Vaccination: a Pillar of Prevention

The existing arsenal of tools for effective treatment (from rehydration to antibiotics) and prevention (from improved sanitation to oral vaccines) should be delivered in the context of comprehensive care to strengthen Haiti's health system. As incidence declines, long-term prevention measures, including rolling out vaccination campaigns and strengthening water and sanitation infrastructure, should be implemented. But prevention should not come at the expense of acute care: the biggest priorities remain case finding and treatment. Rather, additional resources should be secured so that immunologically naïve populations can be vaccinated while acutely ill patients are being treated.

Past epidemics have been curbed without vaccines, but we believe that vaccination has a significant role to play in Haiti given the vulnerability of the post-earthquake health, water, and sanitation systems and the observed virulence of the El Tor strain. Any rational vaccine strategy must be coordinated by local authorities. However, the MSPP is currently wary of NGOs haphazardly delivering vaccine because uneven access could trigger social frictions and interfere with other cholera prevention and treatment efforts. The MSPP has called for nothing less than a universal vaccination campaign—an end goal this document endorses.

If sufficient vaccine and resources were mobilized, large-scale immunization could bring a more rapid end to the current epidemic and help prevent a resurgence of cases. However, there is insufficient vaccine currently available to cover the 10 million people in Haiti (20 million doses); a scale-up strategy that first targets vulnerable groups (like children under 5) is needed. Antiretroviral therapy roll-out in Haiti showed that pilot projects demonstrating the utility of an intervention can boost consensus, political will, resources, and manufacturing for that intervention. Pilot vaccination projects for cholera could do the same.

Recent estimates suggest that 4 million vaccine doses could be available by March 2012, and this figure will only increase as implementation begins (Pan-American Health Organization, Cholera Update Conference Call, February 8, 2011, unpublished data). Indeed, in November 2010, experts estimated 200,000–400,000 doses could be available in 1 year; by early February 2011, they estimated 4 million [S35] [93]. A universal vaccination campaign would require a strategy for timing, coverage, procurement, and the mass action effects of vaccination. Further, scaling up efforts in Haiti would build momentum toward a global stockpile to prevent similar shortages during future outbreaks [94]. Finally, this strategy should include new research on live attenuated vaccines that would confer rapid and long-lived immunity after a single dose (instead of the two doses that existing vaccines require). Such research should not, however, come at the expense of implementation of current vaccines and other components of cholera treatment and prevention; it should be construed as an important part of a comprehensive and long-term response to cholera.

Concerns about the cost-effectiveness and feasibility of implementation have hindered progress on vaccination in Haiti, but these concerns are fading [95]. As with therapeutics for AIDS and multidrug-resistant tuberculosis, the costs of cholera vaccines vary enormously; hence, confident claims about cost-effectiveness should be closely examined [S36] [25], [96]. Implementation bottlenecks are also surmountable: Zanmi Lasante (Partners In Health's sister organization) achieved a 76% completion rate for a three-dose course of HPV vaccine in rural Haiti, and the earthquake occurred between the second and third dose for many of the girls enrolled [S37] [97]. This is almost twice the rate of completion for similar courses in US settings [S38] [98]. Mass two-dose oral cholera vaccination was shown to be feasible in Sudanese refugee camps [S39] [99]. While refugee camps in Sudan pose different management challenges than IDP camps in Port-au-Prince, such vaccination campaigns are an important precedent and should be examined for lessons that may pertain to Haiti.

There are two oral cholera vaccines on the market, both feasible for use in Haiti. Dukoral, produced by Crucell and licensed in over 60 countries, contains recombinant cholera B subunit, which stimulates anti-toxic and antibacterial immunity [S40] [25]. Dukoral has been shown to have roughly 85% protective efficacy among two-dose recipients (over 6 months) in Bangladesh and Peru [100]–[102] and confer protection up to 3 years following vaccination [S41] [103]. Limited data exists on its use in non-endemic zones, but two to three doses appear to be effective [25].

A closely related bivalent oral cholera vaccine is licensed as Shanchol in India and mORCVAX in Vietnam. The latter is produced by VaBiotech and currently intended for domestic use in Vietnam, while Shantha Biotechnics (acquired by Sanofi-Aventis) is applying for WHO prequalification to produce Shanchol for international use. (It is already licensed and commercially available in India.) A 5-year efficacy trial in Kolkata, India, is ongoing, but an interim analysis after 2 years indicated an overall protective efficacy of 67% against culture-confirmed cholera among those who received two doses [S42] [25], [104].

These vaccines have been shown to be effective at preventing both El Tor cholera in endemic settings and the newly emergent El Tor hybrid strains, with protective immunity expected to begin about 1 week after the last dose [S43] [25], [102], [105]–[107]. Observed herd immunity effects (with vaccine coverage rates of 50%) suggest that conventional studies may yield artificially low efficacy estimates [108]. Studies that take herd immunity into account have found cholera vaccination in endemic countries to be cost-effective [S44] [109]. One study in Bangladesh found a dramatic reduction in cholera incidence after widespread vaccination [96]. Mathematical models predict more substantial benefits: one projected that vaccinating just over 50% of the population would lead to a 93% reduction in cholera incidence [S45] [110]; another (published in 2011) projected that 30% coverage would lead to a 55% reduction [111]. Recent scholarship also points to the significant value of reactive (or delayed) vaccine use. A recent case-control study in Hanoi, Vietnam, found a protective efficacy of 76% with reactive use of oral cholera vaccine [112]. Another recent paper suggested that, if widespread vaccination had been launched during epidemics over the last decade, 40% of cases and deaths could have been averted [113]. Although the model does not take the benefits of herd immunity into account, it predicts that reactive cholera vaccination campaigns with 50% coverage would alone have prevented over 10,000 cholera cases in the Zimbabwe epidemic of 2008 and 2009.

Dukoral is manufactured at a cost of US$6 per dose and has a retail price in North America of US$50 to US$75 per dose [114]; Shanchol is manufactured for less than one-third of the cost of Dukoral [114] and is available to developing countries through the public sector at less than US$2 per dose [115]. Large purchases, advance orders, pooled procurement, and other market-based strategies could significantly reduce vaccine cost [S46] [114]. An immunization campaign in Vietnam estimated the cost of a complete mORCVAX series (a bivalent vaccine related to Shanchol), including delivery, at only US$0.89 per person [S47] [107].

While there are currently fewer than 400,000 vaccine doses ready for shipment from their manufacturers [115], advance purchase commitments could increase availability to several million. Past experience underscores the value of publicly ensured purchases [S48]. For example, a demand-side approach to bring a late-stage pneumococcal vaccine to market for use in sub-Saharan Africa and Asia involved an advance market commitment led by the Global Alliance for Vaccines and Immunisation (GAVI). Such funding boosted production, lowered prices, and expanded vaccine access in these regions [116], [117]. Economies of scale also contribute to lower production costs, as observed during the scale up of antiretroviral therapy for AIDS and drugs for other large-scale treatment efforts [118]. All of these factors highlight the benefits of a global stockpile of cholera vaccines.

Models suggest that vaccination campaigns that target vulnerable populations (such as immunologically naïve groups living in crowded conditions with limited access to clean water) can be equally cost-effective as other prevention methods [119]. Existing water and sanitation efforts have helped keep urban cholera rates low and should be continued and scaled up—but not to the exclusion of vaccine delivery. Both robust water systems and vaccination are needed to prevent case resurgence. Vaccination could also help cut costs by limiting the need for future antibiotic use [120].

Finally, existing vaccination and treatment delivery channels provide an infrastructure for cholera vaccine administration. Unlike more complex medical interventions, oral vaccines can be implemented rapidly and effectively by CHWs. A cholera vaccination campaign could therefore leverage existing health worker networks without taking doctors and nurses away from the provision of acute care [S49] [98].

We recognize that there is insufficient vaccine today for an immediate mass campaign, and that the current epidemic could be curbed before such a supply becomes available. Without significant investment in Haiti's weakened health system, there will continue to be insufficient human and financial resources to deliver a mass vaccination campaign. Nonetheless, we believe a rational vaccine strategy should be pursued immediately. Although the 1 million doses available would provide a complete vaccine course to only 500,000 people (about 5% of Haiti's population), targeting vulnerable populations could help to reduce transmission, decrease the likelihood of resurgence, and put gears in motion toward amassing a global stockpile—an outcome that would be beneficial for this epidemic and the next.

### Education and Behavioral Change: Necessary but Insufficient

Coupling community education about cholera transmission with the provision of necessary supplies could improve hygienic behavior and reduce social stigma. Numerous public education campaigns have disseminated information about cholera transmission, diagnosis, and treatment, including the location of treatment sites and transport options. These efforts have targeted populations in slums, IDP camps, schools, and other public fora, urban and rural, around the country. In the first 2 months of the epidemic, camp management teams implemented 670 cholera risk-reduction activities in IDP camps and their surrounding communities; UN education cluster partners distributed cholera prevention and water treatment protocols in schools across the country; phone companies, along with the International Federation of the Red Cross and Red Crescent Societies, the International Organization for Migration, and others, sent public health warnings via SMS; and radio stations dedicated broadcasts to education programs, provided updates from the MSPP, and answered caller questions [89]. All of these efforts should continue to be implemented throughout the country.

Yet the success of these efforts has been limited. Surveys to assess IDP camp residents' awareness of cholera management protocols—where to access clean water, obtain ORS, and dispose of cadavers—reveal serious gaps, particularly concerning where to obtain ORS. Continued surveillance of education programs could help improve message targeting and efficacy [89].

The more important reason for the failure of education and behavioral campaigns is that, like other diseases of poverty, cholera's spread is dictated less by bad behavior than by a chronic shortage of tools and resources. People may defecate near public water supplies if they lack decent sanitation and sewage systems; they may draw water from contaminated sources if there is no alternative; and they may not wash their hands if soap is too expensive or inaccessible. The structures of poverty mediate risk of cholera infection, and therefore information can only keep cholera at bay if vulnerable populations are furnished with necessary resources and supplies.

As noted, proper hand-washing can significantly reduce transmission of cholera and other waterborne diseases. Yet without adequate access to soap and clean water, public health messages about hand-washing are futile. As late as December 7, 2010, UN officials reported soap shortages in IDP camps, urban slums, and rural communities [89]. Therefore, education and behavioral change campaigns must be linked with distribution efforts to make prevention and treatment tools available to all those receiving public health messages. Progress on this front is underway. Oxfam, for example, is using former immunization points—locations familiar to the public—to distribute soap and ORS and to conduct hand-washing and prevention programs. It has brought 40 local organizations into its fold an aims to reach 340,000 beneficiaries with this strategy [89].

Public health messaging has also sought to address cholera-related stigma. Health workers have encountered resistance to building treatment sites because local residents fear that these facilities could bring cholera to the community. OCHA and many Haitian media groups have reported lynchings of individuals thought to have used witchcraft to spread cholera [S50] [89], [121]. Stigma is, in part, tied to ignorance about disease etiology, transmission, prevention, and treatment. But it is equally tied to an absence of tools to control the disease: there was great stigma about AIDS in Haiti until access to antiretroviral treatment transformed the disease from a death sentence into a chronic, manageable condition [55], [122], [123]. The same is likely true for cholera. Behavioral and education efforts must therefore be linked to a flow of resources and medical supplies to be effective.

### Building a Feedback Loop: Surveillance and Monitoring

Laudable efforts are underway to collect and compile surveillance data on the national level and to map health facilities and care capacity. But these efforts must be expanded to avoid a resurgence in cases in the coming months and years. Haiti's existing national surveillance system regularly aggregates reported case data: local MSPP officials send hospital and clinic case counts to central authorities who aggregate and transmit the information to the central government; the MSPP then posts all non-hospitalized and hospitalized cases daily on a public Web site [12]. In addition, geographic information systems (GIS) and basic capacity data have been used to map treatment sites across the country. Reliable data regarding treatment availability (and lack thereof) would improve the delivery of cholera care [89].

Additional sentinel sites and greater resources for surveillance efforts could help anticipate, treat, and prevent the further spread of cholera and other preventable diseases. Surveillance is a key part of treatment and prevention, and simple but effective models exist. For example, a four-symptom diagnostic method (diarrhea, fever, cough, skin rash) has helped identify transmissible diseases in one IDP camp [S51] [124]. Robust individual case data also illuminates new information about epidemiology, prevention and treatment efficacy, and the differential vulnerability of populations. Surveillance enables treatment experiences to drive prevention strategies and guides the efficient allocation of limited resources.

That the country's early warning system was able to rapidly detect the first cases of cholera highlights the strong leadership of the MSPP, PAHO, and the CDC. However, some have suggested that cases are being underreported, particularly in rural areas. For example, many children die before they reach treatment sites, and therefore their deaths may not be captured by current monitoring techniques (Dr. Jean-William Pape, personal communication). Poor coordination among health providers has further hindered the MSPP's case data collection [6]. To supplement the existing reporting system, mathematical models can be useful for understanding the changing nature of disease distribution [S52] [125], [126].

Cholera is likely to become endemic in Haiti, and it presents a threat to other countries in the region and around the world. Surveillance must be part of a comprehensive response to the immediate epidemic and a cornerstone of the country's health infrastructure. Education, supply chain, treatment, and prevention channels can be coordinated to feed information to a central source; early results of antibiotic scale-up and vaccine pilots should guide future implementation; the MSPP should coordinate all monitoring to strengthen its national surveillance systems for cholera and other notifiable diseases.

## Conclusion. Rapid Implementation and the Road to Health Systems Strengthening

The comprehensive, integrated strategy described in this document should be implemented to mitigate the current cholera epidemic and strengthen the Haitian health system in the long term. Scaled-up delivery of ORS, intravenous therapy, antibiotics, vaccine, soap, and other tools for cholera prevention and care should be linked to the provision of primary health care services to strengthen supply chains countrywide. CTCs, CTUs, ORPs, and other cholera-specific facilities and infrastructure should be integrated into the public health system. We can build on the extensive national HIV treatment network to establish permanent cholera treatment sites at hospitals and health centers. Cholera caregivers should also be trained to identify and treat other diarrheal diseases and illnesses of poverty. In other words, cholera-specific interventions should be used as a wedge to bolster primary health care services and strengthen the Haitian health system.

### Towards an Implementation Plan

The Haitian government must lead the ongoing response to cholera in order to promote collaboration between the multiplicity of donors, aid groups, and NGOs offering assistance in Haiti—all of which have disparate protocols and goals. Even with the best intentions, these groups can impose significant burdens on a stressed public health system. Only a well-coordinated response with clear leadership from the MSPP and DINEPA will be able to address the acute challenges of the current outbreak while also building robust health, water, and sanitation systems to prepare for future epidemics.

The goal of this paper is to highlight the priorities of a comprehensive, integrated, and long-term cholera response; the next and even more important goal is the implementation of this strategy. Implementation will require political will and substantial, long-term financial commitments from global health authorities, donors, and health providers. In a December 2010 report to the UN General Assembly, Secretary-General Ban Ki-moon asked nations to help Haiti respond to the cholera epidemic [S53] [127]. As of February 4, 2011, only 45% of the US$175 million appeal had been met [45]. Haiti needs long-term financial support or it will still be vulnerable when the next epidemic—of cholera or another preventable disease—strikes.

### A Call for Consensus and Action

The months following the earthquake on January 12, 2010, were among the most trying in Haiti's history. Continued international assistance can prevent suffering and death in the near term and help Haiti to rebuild better in the long term. But the response to cholera must be comprehensive, linking prevention to care and coordinating Haitian and non-Haitian partners. In the last two decades, a period of economic stagnation and frequent political turmoil, comprehensive and integrated community-based prevention and care reduced infant mortality rates in Haiti by two-thirds and cut the AIDS epidemic in half. This model has since been adopted across the developing world. We have the opportunity to do the same with cholera.

Debates about prevention versus care are misguided. We must integrate population-based prevention measures with medical treatment to contain the outbreak and save thousands of lives. Haiti faces both urgent and entrenched challenges that require solutions combining speed in the short-term and sustainability in the long-term.

Our objectives in Haiti should be no different than those we would set in the Dominican Republic, the United States, or any other neighboring country. This “Haitian” epidemic is part of a global pandemic that claims over 100,000 lives each year. The presence of cholera in Haiti highlights the volatility of transmission in an era of global migration and trade, and it threatens to spread the pathogen in the Caribbean and across the Western Hemisphere. All nations share a common interest in eliminating cholera wherever it occurs: a world in which the burden of cholera follows the burden of poverty is not just inequitable, it is dangerous, even to rich nations.

We, a group of 44 medical and public health researchers, policymakers, funders, and practitioners, working for research universities, government agencies, NGOs, international multilateral organizations, and the private sector in Haiti, the US, and many other countries around the world, strongly support a comprehensive, integrated, and long-term response to cholera in Haiti. We stand behind the government of Haiti and are committed to helping implement this strategy together and in the months and years ahead.

## Supporting Information

Text S1 Supplemental references.
(DOC)
