# Supplementary material for: Meeting Cholera's Challenge to Haiti and the World: A Joint Statement on Cholera Prevention and Care
Source: PLoS Negl Trop Dis. 2011 May 31;5(5):e1145. doi: 10.1371/journal.pntd.0001145 (PMC3104956; doi:10.1371/journal.pntd.0001145)
Supplement: Text S1 — Supplemental references. (DOC) [file pntd.0001145.s001.doc]

[S1] A 2007 WHO report estimates Haiti’s maternal mortality rate at 1,700 deaths per 100,000 women of reproductive age in 2005 (“Maternal Mortality in 2005.” Geneva: WHO, 2007. Available: <http://www.who.int/whosis/mme_2005.pdf>). The United Nations’ World Population Prospects Report (New York: United Nations, 2007) estimates Haiti’s infant mortality rate at 48.8 per 1,000 live births from 2005-2010.

[S2] For example, Jamaica’s cholera epidemic of 1850-51 killed around 30,000 persons but never reached Haiti. See, e.g., Kiple K . Journal of Latin American studies 17: 1; 157-177, 1985; Ackers ML et al. Int J. Epidem. 27: 330-334, 1998.

[S3] Cases have been documented in the Dominican Republic, Venezuela, and the United States.

[S4] Further study of the location, time, and nature of the first cases is needed to better understand the origin and spread of the outbreak.

[S5] The MSPP reported an average case-fatality rate of 7% as of October 30, 2010. See the PAHO EOC Situation Report # 9 (November 1, 2010), available: http://new.paho.org/disasters/index.php?option=com_docman&task=doc_download&gid=1374. Case fatality rates in previous epidemics of the last decade in Zimbabwe, Kenya, Nigeria have ranged from 2-4%. Harris J, LaRocque R, Charles R, Mazumder R, Khan A, et al. (2010) Cholera’s western front. *Lancet* 376: 1961-1965.

[S6] In the first 48 hours, the case fatality rate at PIH/ZL/MSPP facilities was as high as 10%, though it subsequently dropped to 2% once the health care infrastructure was able to respond and patients arrived sooner. Walton D, Ivers L (2011) Responding to cholera in post-earthquake Haiti*.* *New England Journal of Medicine* 364: 3-5.

[S7] Between 2008 and 2009, Zimbabwe experienced a nearly yearlong epidemic which took 4,500 lives and spread to Zambia and South Africa. Evidence suggests that political instability contributed to underreporting of cases and deaths in Zimbabwe. See, e.g., Mintz E, Guerrant R (2009) A lion in our village—the unconscionable tragedy of cholera in Africa. *N*ew *England Journal of Medicine* 360: 1060-1063.

[S8] For example, some have suggested that childhood cholera cases are underreported. Of the 124,482 reported hospitalized cases as of February 8, 2011, only 8.8% were children under 5 years of age (10,994 cases) even though they are expected to be more vulnerable to cholera infection. See USAID Fact Sheet #20 (February 18, 2011), available: <http://www.usaid.gov/our_work/humanitarian_assistance/disaster_assistance/countries/haiti/template/fs_sr/fy2011/haiti_ch_fs20_02-18-2011.pdf>. One explanation for this low percentage is that many sick children die before they can reach a care center, and their deaths may go unreported.

[S9] In the north, MSF was receiving 380 patients daily and 1,100 every day in early December 2010 (Medecins Sans Frontieres, 2010 December 9). In Haiti’s rural north, the cholera rate at this time showed only the ‘tip of the iceberg’ as the outbreak spread. Available: <http://www.msf.org/msf/articles/2010/12/in-haitis-rural-north-cholera-rate-shows-the-tip-of-the-iceberg-as-outbreaks-spreads.cfm. Accessed 2010> Dec 22.

[S10] Some studies also indicate that the proportion of “severe” cases may be underestimated in El Tor epidemics. Bart K, Huq Z, Khan M, Mosley W (1970). Seroepidemiologic studies during a simultaneous epidemic of infection with El Tor Ogawa and classical Inaba *Vibrio cholerae*. *The Journal of Infectious Diseases* 121:17-24. This paper compares the spectrum of illness for El Tor and classic biotype strains of V. cholerae. The classic biotype produced a lower proportion of asymptomatic infections (59% vs. 75%) and a higher proportion of severe infections (11% vs. 2%) than the El Tor biotype. Some have hypothesized that the spectrum of variant El Tor biotype strains with classic toxin type would more closely resemble that of the classic biotype, though evidence is only anecdotal to date.

[S11] The CDC update from December 24, 2010 reported a total of 59 reported cases in the Dominican Republic and 5 cases in Florida. Centers for Disease Control and Prevention (2010) Update on cholera—Haiti 2010. *Morbidity and Mortality Weekly Report* 59: 1637-1641. Available: <http://www.cdc.gov/mmwr/preview/mmwrhtml/mm5950a1.htm?s_cid=mm5950a1_w>. Accessed 2011 Jan 8. Ministerio de Salud confirma casos de colear en el pais. (2011 January 26) Available: <http://www.globovision.com/news.php?nid=176062>. Accessed 2011 Feb 4. In addition, cases have been reported in Massachusetts. Smith S (2011 January 29) 2d cholera case confirmed. *Boston Globe* Available: <http://www.boston.com/news/local/massachusetts/articles/2011/01/29/massachusetts_confirms_its_second_cholera_case/>. Accessed 2011 Feb 5.

[S12] This quotation is attributed to Joia Mukerjee, M.D., the medical director of Partners In Health, a nongovernmental organization that has helped provide cholera prevention and treatment services in Haiti, in Fraser, B (2010) Haiti still gripped by cholera as election looms. *Lancet* 376: 1813-1814.

[S13] In 1980 Pape et al introduced oral rehydration therapy at the State University Hospital (HUEH) in Haiti, which, along with other interventions, contributed to a reduction of in-hospital mortality from over 44% to less than 1% within the year. See Pape JW, Balasubramanyan R, and Rohde JE. Intestinal Illness in *Medical Care of Refuges*. R.A. Sandler and T.C. Jones (eds.), New York Oxford University Press, 1988, pp. 364-3. Within one year, a national program was created with the Cornell-GHESKIO unit as the national training center; 14,000 physicians, nurses, auxiliary nurses were trained over a 10-year period with the establishment of Rehydration Units in each public hospital department. GHESKIO mobile teams made monthly visits to each site to accompany recently trained individuals. The model depended most of all on mothers: during the 3-day in-patient stay, mothers received basic training about hygiene and sanitation, dehydration diagnosis, and ORT provision. As the community became informed about the use of ORS, the number of children admitted to the Cornell-GHESKIO unit decreased sharply from over 7,000 per year (4 children per beds) to less than 200 in 2000 and the unit was closed. This intervention contributed to the two-third decrease in national infant mortality rate that occurred despite the arrival of AIDS and worsening socioeconomic conditions over the last 3 decades. The Demographic and Health Surveys confirm a sizeable reduction in childhood mortality in the last 25 years: from 1980-1984, infant mortality was found to be 120 per 1000 live births and under-five mortality was 176 per 1000 live births; in 2005-2006, these had dropped to 57 and 31, respectively. See Demographic and Health Survey (1994-1995) Haiti Survey on Mortality, Morbidity, and Utilization of Services Summary Report (Available: at http://www.measuredhs.com/pubs/pdf/SR49/SR49.pdf); Demographic and Health Survey (2005-2006) Enquête Mortalité, Morbidité et Utilisation des Services Rapport de synthèse (Available: at http://www.measuredhs.com/pubs/pdf/SR124/SR124.pdf). Encouraging results such as these show that a similar approach could be successful in the fight against cholera in Haiti. It is because of this experience in the prevention and treatment of diarrheal diseases that GHESKIO has been able to mount a swift response by setting up some of the first CTCs in the West Department.

[S14] Zinc supplementation has been shown to reduce the severity and duration of diarrhea in children under 5 years of age. Short course zinc supplementation reduces the incidence of diarrhea for 2 to 3 months. The WHO recommends that 10-20 mg/day of zinc be given for 10 to 14 days to all children with diarrhea.

World Health Organization (2005) The treatment of diarrhea: A manual for physicians and other senior health workers. Page 5. Available: <http://whqlibdoc.who.int/publications/2005/9241593180.pdf>. Accessed 2010 11 Dec. A more recent publication indicates that 5 days of treatment can be as efficacious as the 10 day treatment in preventing diarrhea in the subsequent three months among children in Bangladesh. This finding reduces cost and increases compliance Alam DS,Yunus M, Arifeen S, Chowdury H, Larson C, et al (2011) Zinc treatment for 5 or 10 days is equally efficacious in preventing diarrhea in the subsequent 3 months among Bangladeshi children. *J Nutr* 141:312-315. While the exact mechanism is still not understood, improved transport of electrolytes with zinc may lead to reduced stool output and duration, explaining the mechanism of zinc supplementation. Roy SK, Hossain M, Khatun W, Chakraborty B, Chowdhury S, et al (2008) . Zinc supplementation in children with cholera in Bangladesh: randomized controlled trial. *BMJ* 2008; 336: 266-268.

[S15] In addition to vitamin A, WHO recommends folate, copper, zinc, and magnesium for children with diarrhea. World Health Organization (2005) The treatment of diarrhea: A manual for physicians and other senior health workers. Page 5. Available: <http://whqlibdoc.who.int/publications/2005/9241593180.pdf>. Accessed 2010 Dec 11. Page 25.

[S16] Epidemiological models can implicate the broader effects of antibiotic use on the epidemic and the healthcare system.

[S17] A total of 101 CTCs, 185 CTUs, and 927 ORPs are operational nationwide as of February 4, 2011. United Nations Office for the Coordination of Humanitarian Affairs. Haiti Cholera Situation Report #35. February 4, 2011. Available: [http://haiti.humanitarianresponse.info/Portals/0/OCHA%20Haiti%20_Cholera%20Sitrep_35.pdf](http://haiti.humanitarianresponse.info/Portals/0/OCHA Haiti _Cholera Sitrep_35.pdf). Accessed 2011 Feb 10.

[S18] The stress on rural communities hosting hundreds of thousands of people displaced by the January 2010 earthquake has been frequently overlooked. Their sanitary and medical systems are stretched to the limit. Roads are poor, and there are few medical providers of any stripe in these regimes. Yet several exceptions do exist. From the immediate aftermath of the earthquake through the cholera response today, the Cuban medical brigade—the largest from any country—has sought out some of the most vulnerable, rural communities. Cuban doctors now contribute to treatment for about 40% of cholera cases across Haiti. Doctors Without Borders and other humanitarian groups have also provided technical expertise. There are nurses in the Haitian diaspora, who could help improve the quality of care. Many present after the earthquake have left, but many also remain in Haiti and could help implement a more ambitious prevention-and-care plan.

[S19] On November 15-16, a two-day train-the-trainer program in Port-au-Prince was held to educate healthcare providers in Haiti on cholera treatment and management techniques. Centers for Disease Control and Prevention (2010) Update: cholera outbreak—Haiti 2010. 59: 1473-1479. Available: <http://www.cdc.gov/mmwr/preview/mmwrhtml/mm5945a1.htm. Accessed 2010 Dec 5>.

[S20] Private sector water projects date back to the 1970s when tankers transported water from wells and other public sources to sell to cistern owners by the bottle.

[S21] These figures likely overstate access to improved water sources, as public systems are rarely able to provide water during the dry season. UNDP (2004), La vulnérabilité en Haïti: Chémin inévitable vers la pauvreté? Rapport national sur le développement humain – Haïti.

[S22] Survey data showed that for just 3 buckets of water in a day, Haitians spend more than 10% of the median household expenditure for all basic necessities, including food, water and charcoal. Varma M, Satterthwaite M, Klasing A, Shoranik T, Jean J, Barry D, et al (2008) Wòch nan Soley: The Denial of the Right to Water in Haiti. Francois-Bagnoud Center for Public Health. page 75. Available: www.pih.org/page/-/reports/Haiti_Report_FINAL.pdf . Accessed 2010 Dec 5. Regarding contamination, a recently published study found cryptosporidium (the most common agent associated with diarrhea in AIDS patients) in 91% of reservoirs, 54% of public standpipes, and 100% of surface waters. See Pape JW. Treatment of gastrointestinal infections. *AIDS* 1989;2:161-7; Bras A, Evans E, Obison L, Brasseur P, Pape JW, Raccurt C. Biological 6 Assessment of cryptosporidium oocysts in drinking water in Port-au-Prince , Haiti, Env, Risques et Santé, 6: 5, 2007.

[S23] In addition, adding adequate hypochlorite disinfectant to water used to prepare ORS, and waiting 30 minutes before adding the packaged salts, ensures that bacteria will be inactivated before ORS is added. Daniels NA, Simons SL, Rodrigues A, Gunnlaugsson G, Forster TS, et al (1999). First do no harm: making oral rehydration solution safer in a cholera epidemic. *American Journal of Hygiene and Tropical Medicine* 60: 1051-1055.

[S24] For example, some studies estimate household interventions (47% reduction in disease) to be more effective than improved wells, boreholes, and communal stand pipes (27% reduction in disease). Clasen T, Schmidt WP, Rabie T, Roberts I, Cairncross S (2007). Interventions to improve water quality for preventing diarrhea: systematic review and meta-analysis. *BMJ* 334: 782.

[S25] Examples include Tata’s Swach (about US$20), the Vestergaard Straw Family Filter (also approximately US$20), and the Basic Water Needs Foundation Tulip filter (about US$10). The Tulip water filter, available: [http://www.basicwaterneeds.com/grove2/fmf/userfiles/E-info%20Tulip%20water%20filter%20November%202010.pdf](http://www.basicwaterneeds.com/grove2/fmf/userfiles/E-info Tulip water filter November 2010.pdf). Accessed 2010 Dec 12. For example, the Pureit water treatment unit (developed by Hindustan Lever Limited) combines filtration with chlorination. It is a gravity-fed device that filters and chlorinates water of unknown microbial content without using electricity, providing about 9 liters of drinkable water in 2-3 hours and offering a mechanism for safe water storage. The carbon block pre-filter, chlorine dispenser, and polisher can treat up to 1500 liters before they need to be replaced. In India, the unit costs approximately US$35; a set of consumables is slightly less than US$6. The average cost of a unit is approximated at US$0.06 per day for a household using 10 liters/day. Clasen T, Nadakatti S, Menon S (2006). Microbiological performance of a water treatment unit designed for household use in developing countries. *Tropical Medicine and International Health* 11: 1399-1405.

[S26] The approximate cost of PuR to consumers is less than $0.01 per liter. Procter & Gamble provides PuR at-cost ($0.035 per sachet) to relief agencies and social marketing groups. The annual cost of PuR is estimated at approximately $11 per user at the consumer cost and $3.83 per user at the relief cost (estimate based on the consumption of 3 liters of treated water per day Clasen T, Lantagne D (2009). Point of use water treatment in emergency response. Available: <http://pdf.usaid.gov/pdf_docs/PNADS134.pdf. Accessed 2011 Jan 10>.

[S27] Examples include Water Maker (made in South Africa), Bishan Gari (Ethiopia), Thanh Mai (Vietnam), and PuR Purifier of Water (Procter & Gamble). A PuR sachet is a packet that contains two substances in powder form: ferric sulfate (a flocculant) and calcium hypochlorite (a disinfectant). The process of purification first requires one satchet to be poured into a container of 10 liters of water. The water is then filtered through cloth filtration. The hypochlorite destroys the microorganisms in about 20 minutes, after which, the water is ready to drink. Souter PF, Cruickshank GD, Tankerville MZ, Keswick BH, Ellis BD, et al. JD (2003) Evaluation of a new water treatment for point-of-use household applications to remove microorganisms and arsenic from drinking water. *J Water Health* 1:(73-84).

[S28] Sachets that combine flocculation and disinfection agents in powder form mimic the process used by water purification plants, simultaneously ridding the water of particles and harmful organisms. While sodium hypochlorite is used widely throughout the developing world to treat drinking water at the point of use, its efficacy is greatly reduced when water contains high concentrations of organic matter. Large doses placed in turbid water can also produce potentially toxic compounds.

[S29] There are several sachets on the market, some of which have been shown to reduce waterborne cysts by more than 99.9%, viruses by more than 99.99% and bacteria by more than 99.99999% even in highly turbid waters in field studies and emergency situations. PUR Drinking water purification supplies, available: <http://www.butylproducts.co.uk/Products-Services/AidEquipment/Purification/WaterPurificationPUR/> Accessed 5 May 2011.

[S30] Fewtrell and colleagues (2005) conducted a systematic review of 15 intervention studies which shows that household-based water treatment and safe storage was associated with a 35% reduction in diarrheal disease compared to a statistically insignificant 11% for conventional source-based interventions. Fewtrell L, Kaufmann R, Kay D, Enanoria W, Haller L , Colford J (2005) Water, sanitation, and hygiene interventions to reduce diarrhea in less developed countries: a systematic review and meta-analysis. *Lancet* *Infect. Dis*. 5:42-52.

[S31] See “Education and behavioral change: necessary but far from sufficient” (especially p. 44) for an extended discussion about the importance of soap distribution to accompany public-health messaging about hand-washing.

[S32] According to WHO guidelines for treatment of diarrheal illness outbreaks, ventilated pit latrines should be constructed at least 30 meters from drinking water sources, and when possible at least 6 meters away from homes. They should also be downhill from water sources and construction should take into account local factors such as soil type and population density. Maintenance is also required—the slabs and floor should be washed daily and disinfected regularly with cresol or bleaching powder. After a pit is filled to two-thirds capacity (1.3 meters in height), it should be filled with soil and compacted, and a new pit should be dug. World Health Organization (1993) Guidelines for Cholera Control, Annex 1: Building a ventilated improved pit latrine. Available: <http://helid.digicollection.org/en/p/printable.html. Accessed 2011 Jan 5>.

[S33] Active surveillance of cholera outbreaks was assessed in a study conducted in Peru between 1993 and 1996. The severity of outbreaks was found to be 7.6 times greater when *V. cholerae* O1 was detected in sewage water (and 2.4 times greater when cholerae O1 bacteriophages, or vibriophages, were present) up to four weeks prior to the outbreak. This study illustrates the benefit of active surveillance of cholera in predicting the severity of cholera outbreaks. Madico G, Checkley W, Gilman RH, Bravo N, Cabrera L, et al. (1996) Active surveillance for Vibrio cholerae O1 and Vibriophages in sewage water as a potential to predict cholera outbreaks. *Journal of Clinical Microbiology* 34: 2968-2972.

[S34] PAHO director Jon Andrus identifies cadaver disposal as a key issue. PAHO has been involved in deploying experts in the area to Haiti. Pan American Health Organization. (2010 October 26) Cholera cases likely to increase in Haiti. Available: <http://www.reliefweb.int/rw/rwb.nsf/db900sid/LSGZ-8ALH2Z?OpenDocument&query=haiti>. Accessed 2010 Dec 11.

[S35] In particular, experts from the International Vaccine Initiative and PAHO anticipate 3 million doses of Dukoral and 1 million doses of Shanchol could be ready by March of 2012. Pan American Health Organization. Cholera Update Conference Call. February 8, 2011.

[S36] Several examples exist of varying cost-benefit analyses. The differing prices of Dukoral vs. Shanchol are one of several factors that greatly alter overall production costs. Ali et al.’s 2005 reanalysis of the indirect effects (herd immunity) of cholera vaccination in Bangladesh suggests additional benefits that may not be captured by cost-benefit analyses. Ali M, Emch M, Seidlein L, Yunus M, Sack D, Rao M, Holmgren J, Clemens J (2005). Herd immunity conferred by killed oral cholera vaccines: a reanalysis. *Lancet* 366: 44-49). The WHO vaccines position statement notes, “There is a lack of cost- effectiveness analyses of the synergistic impact of vaccination when added to traditional public health interventions against cholera, such as providing health education and improving water and sanitation.” World Health Organization (2010) Cholera vaccines: WHO position paper. *Wkly Epidemiol Rec* 85: 126.

[S37] Unpublished data from PIH’s project in Haiti shows remarkably high success of HPV administration in Haiti despite the damage of the earthquake. Partners In Health (2010 August 27) Curbing cervical cancer in Haiti. Available: <http://www.pih.org/news/entry/curbing-cervical-cancer-in-haiti/>. Accessed 2010 Dec 5.

[S38] According to the US CDC, as of August 2010, only 27% of girls aged 13-17 completed 3 doses of HPV vaccine. Centers for Disease Control and Prevention (2010 August 19) Teen vaccination rates increasing across the US. Available: <http://www.cdc.gov/media/pressrel/2010/r100819b.htm>. Accessed 2010 Dec 5.

[S39] 63,220 doses of vaccine were administered among 44,000 Sudanese refugees, with 83.0% and 75.9% coverage for the first and second rounds, respectively. Overall cost of the campaign, excluding the vaccine, amounted to US$14,655, and the vaccine was administered (at best) at 200 doses per vaccination site per hour. Legros D, Pacuet C, Perea W, Marty I, Mugisha NK, et al. (1999) Mass vaccination with two-dose oral cholera vaccine in a refugee camp. *Bull World Health Organ.* 77: 837-842.

[S40] Dukoral is recommended in 2 oral doses for those older than 6 years and in 3 doses for children 2–5 years, with an interval of 1–6 weeks between doses. It is not licensed for children under the age of 2. Studies suggest that it remains stable at 37 degrees C for up to one month (though its label calls for storage at 2-8 degrees C), and it is administered with a buffer and reconstituted in water. World Health Organization (2010) Cholera vaccines: WHO position paper. *Wkly Epidemiol Rec* 85: 121.

[S41] Clemens et al. found in a randomized double-blind trial among children aged 2-15 years and women over 15 in Bangladesh that protective efficacy for both whole cell recombinant/B subunit and whole-cell vaccine types in El Tor cases was 39% and 40%, respectively. Clemens JD, Sack DA, Harris JR, Loon F, Chakraborty J, et al. (1990) Field trial of oral cholera vaccines in Bangladesh: results from three-year follow-up. *Lancet* 335: 270–273.Lancet.

[S42] Shanchol is administered in 2 doses, 14 days apart, for all individuals except infants under one year old. Unlike Dukoral, it does not require buffer or reconstitution in water. The vaccine has been found to be protective in all age groups, including children aged 1–4 years, and the protection showed no decline during the second year of follow-up. World Health Organization (2010) Cholera vaccines: WHO position paper. *Wkly Epidemiol Rec* 85: 125...Data from: Abramson JS, Bhutta Z, Clemens J, DeRoeck D, Henkens M, et al. (2009) Background paper on the integration of oral cholera vaccines into global cholera control programs. Draft document to be presented to WHO SAGE in October 2009. 5 p. Available: <http://www.who.int/immunization/sage/1_Background_Paper_Cholera_Vaccines_FINALdraft_13_oct_v2.pdf>. Accessed 2011 Jan 20.

[S43] Dukoral has been shown to be effective against classical cholera, as well. While Shanchol has never been evaluated against classical cholera, the constituent 01 serogroup strains of Shanchol are the same as those in Dukoral, albeit in higher concentrations. Thus, it is reasonable to speculate Shanchol would also be protective against classical cholera. World Health Organization (2010) Cholera vaccines: WHO position paper. *Wkly Epidemiol Rec* 85: 117-128.

[S44] Incidence was 7/1000 in neighborhoods where vaccine coverage was less than 28%, and dropped to 1.47/1000 in neighborhoods with coverage greater than 51%. Jeuland et al. evaluated mORC-VAX as cost-effective when herd protection was accounted for in data from vaccination programs in Kolkata, India, and Beira, Mozambique. Jeuland M, Cook J, Poulos C, Clemens J, Whittington D (2009). Cost-effectiveness of new-generation oral cholera vaccines: a multisite analysis. *ISPOR* 12: 899-908.

[S45] This study also took into account both direct protection of vaccine recipients and indirect benefits for the broader community. Longini IM, Nizam A, Ali M, Yunus M, Shenvi N, et al. (2007) Controlling endemic cholera with oral vaccines. *PLoS Medicine* 4: e336.

[S46] Frew et al. estimates Shantha Biotechnic’s production cost at <$2/day. Frew SE, Liu V, Singer P (2009) A Business Plan to Help the ‘Global South’ in its Fight Against Neglected Diseases. *Health Affairs* 28: 1764.

[S47] In this mass immunization, the vaccine price accounted for 79% and delivery for 21% of this total cost estimate. Thiem V, Hossain M, Son ND, Hoa NT, Rao MR, et al. (2003) Coverage and costs of mass immunization of an oral cholera vaccine in Vietnam. *Journal of Health Population and Nutrition* 4: 304-308.

[S48] In the case of multidrug-resistant tuberculosis, the Green Light Committee negotiated with producers to obtain bulk supplies for distribution to community-based programs around the world.

[S49] Unpublished data from PIH’s project in Haiti shows remarkably high success of HPV vaccine administration in Haiti despite the damage of the earthquake (2010 August 27) Partners In Health. Curbing cervical cancer in Haiti. Available: <http://www.pih.org/news/entry/curbing-cervical-cancer-in-haiti/>. Accessed 2010 Dec 5.

[S50] Intervention teams have dedicated efforts to psychosocial concerns through community messaging, support groups, and counseling, to complement prevention and treatment efforts. United Nations Office for the Coordination of Humanitarian Affairs (2010 December 7) Haiti: Cholera situation report no. 24. Available: <http://www.reliefweb.int/rw/rwb.nsf/db900sid/MCOI-8BXGAJ/$File/full_report.pdf>. Accessed 2010 Dec 21.

[S51] GHESKIO initiated an active surveillance system based on these four symptoms on the day after the earthquake. Thirty cases of active TB, as well as more than 500 cases of diarrhea, have been diagnosed and treated at the camp clinic. This simple system could be extended to other IDP camps and major slums. See Pape JW, Deschamps, M.M, Ford H, Joseph P, Johnson WD, Fitzgerald D. The GHESKIO Refugee Camp after the Earthquake in Haiti — Dispatch 2 from Port-au-Prince; New Engl J Med. 2010 10 (1056) 1-2.

[S52] For example, as noted, initial statistics underestimated the number of cases and the rate of spread. Walton D, Suri A, Farmer P. (2011) Cholera in Haiti: Fully Integrating Prevention and Care. Ann Intern Med. 2011 Mar 7. [Epub ahead of print]; Andrews J, Basu S. Transmission dynamics and control of cholera in Haiti: an epidemic model. Lancet - 16 March 2011 DOI: 10.1016/S0140-6736(11)60273-0.

[S53] By December 3^rd^, only 20 percent of the need had been met, falling $164 million short of the amount requested by Secretary-General Ban Ki Moon. Associated Press (2010 December 3) Haiti cholera epidemic ‘getting worse by the day.’ Available: <http://www.msnbc.msn.com/id/40494618/ns/health-infectious_diseases/>. Accessed 2010 Dec 6.
